# Treatment of post-resuscitation cicatricial tracheal stenosis after suffering severe COVID-19 associated pneumonia: A report of 11 cases

**DOI:** 10.1016/j.rmcr.2022.101768

**Published:** 2022-10-25

**Authors:** Evgeniy Topolnitskiy, Timofey Chekalkin, Ekaterina Marchenko, Alex Volinsky

**Affiliations:** a Laboratory of Medical Materials, National Research Tomsk State University, Tomsk, Russia; b Department of Surgery, Siberian State Medical University, Tomsk, Russia; c R&D Center, TiNiKo Co., Osong, South Korea; d Department of Mechanical Engineering, University of South Florida, Tampa, USA

**Keywords:** Cicatricial tracheal stenosis (CTS), Tracheomalacia, Circumferential tracheal resection (CTR), Tracheoesophageal fistula (TEF), COVID-19 associated pneumonia, SARS-CoV-2

## Abstract

**Objective:**

Despite the full range of anti-epidemic measures, the rapidly mutating SARS-CoV-2 continues to spread worldwide, causing respiratory and pulmonary pathologies. So far, there are no generally accepted clinical guidelines for treating post-resuscitation cicatricial tracheal stenosis (CTS) after COVID-19 associated pneumonia. This study sought to evaluate the clinical outcomes of surgical treatment and perioperative management of patients who developed CTS.

**Methods:**

A cohort of eleven working-age patients (eight women and three men) with CTS were treated surgically after undergoing invasive artificial ventilation ranging from 5 to 130 days. Along with scarring changes in the tracheal wall, tracheomalacia was noted in five (55.6%) individuals. Circumferential tracheal resection (CTR) with subsequent anastomosis, tracheolaryngeal reconstruction, and endoscopic methods were modalities used to restore airway patency. In cases of CTR combined with tracheoesophageal fistula (TEF), CTR was performed with dissection of the pathological stoma.

**Results:**

In 80% of the cases, CTS was located at the larynx and cervical trachea level. All patients managed to restore adequate breathing through their natural airways with preserved vocal function. No lethal outcomes were observed in the post-op period. Patient outcomes after CTR were considered excellent in nine patients who continued an active lifestyle and went straight to work. One patient, after laryngotracheoplasty and tracheal stenting, is at the final stage of treatment.

**Conclusions:**

These patients are at high risk of developing CTS and require dynamic monitoring. CTR allows early rehabilitation of patients with the best functional outcome. If CTR is contraindicated, laryngotracheoplasty also allows adequate debridement of the tracheobronchial tree and respiratory support.

## 1 Introduction

The rate of post-intubation tracheal stenosis (PITS) varies from 10% to 22%, according to the studies [1]. A longitudinal population-level epidemiological study reported that post-intubation tracheal stenosis is a rare event, with an estimated incidence of 4.9 cases per million per year. However, the absolute number of patients is very significant.

Prior to the pandemic and the widespread of the SARS-CoV-2 virus, significant progress was made in reconstructive surgery for post-resuscitation cicatricial tracheal stenosis (CTS). These surgeries have become available and safe in specialized surgical centers and thoracic facilities, where personnel receive specialized training in tracheal surgery. This fact was proven by the positive outcomes of surgical treatment for CTS, confirmed by the lowest post-op complication rate and mortality along with very rare recurrence. The choice of CTS surgery type and the curative procedure algorithm in usual cases were largely regulated and standardized [[2], [3], [4], [5], [6], [7]].

However, events in Wuhan (China) in December 2019 and the associated well-known tragic consequences for the global healthcare system led to the simultaneous appearance of a large number of patients requiring resuscitation aids and invasive mechanical ventilation (IMV) [[8], [9], [10], [11], [12], [13]]. The most frequent and severe complication in COVID-19 patients is acute respiratory distress syndrome requiring oxygen therapy and respiratory support. Data from China indicate that between 9.8% and 15.2% of patients required invasive ventilatory support [14].

This fact naturally caused an increase in the absolute number of CTS patients. Despite the entire range of anti-pandemic measures, the success achieved in the treatment of the new coronavirus infection, and the emergence of specific vaccines, the COVID-19 pandemic is still ongoing, and SARS-CoV-2 circulating variants continue to spread globally. In turn, the global medical community continues accumulating clinical background, studying the features of diagnosis and perioperative management of patients with causative respiratory pathologies after suffering COVID-19 associated pneumonia. This also applies to CTS and tracheoesophageal fistula (TEF) due to prolonged mechanical ventilation using an orotracheal/tracheostomy tube amid severe conditions [[15], [16], [17], [18], [19], [20], [21]].

In the search for better surgical options, this clinical experience of correction for CTS can be considered by clinicians managing injuries and aftereffects that arose as a result of IMV in the treatment of COVID-19 patients, as well as the distinctive features of perioperative management of severe cases. This work aims to evaluate the clinical outcomes and suggests perioperative care in the surgical management of CTS patients after severe COVID-19 pneumonia and invasive mechanical ventilation. Although the Laryngotracheal Stenosis Committee of the European Laryngological Society sounded the alarm regarding the potential risk of a rising number of COVID-19 related PILS cases, the prevalence of COVID-19 patients who go on to develop PILS remains unknown [13,17].

We herein report 11 patients presenting with this unique and relatively unfamiliar phenomenon during the recovery phase of COVID-19. The objective was to evaluate the clinical results produced by the perioperative care and surgical technique performed in patients with post-intubation tracheal stenosis due to COVID-19 complications.

## 2 Materials and methods

From January 2021 to March 2022, eleven patients (three men and eight women) diagnosed with CTS were treated after undergoing IMV and COVID-19 associated pneumonia. Retrospectively, clinical history in all confirmed cases of COVID-19 included the therapeutic modality pursuant to the WHO guidelines relevant for 2020-21. All COVID-19 patients in the cohort received etiological, pathogenetic, and symptomatic care. The primary approach was preventive measures to avoid a specific symptomatic complex of life-threatening conditions, namely pneumonia, acute respiratory distress syndrome, and sepsis. As part of the medical care, each patient was monitored to control signs of deteriorated clinical conditions. Treatment of concomitant diseases and complications was performed following clinical guidelines and accepted standards for these diseases.

A viral respiratory disease caused by the SARS-CoV-2 virus and complicated with multilobar bilateral pneumonia was confirmed by the typical clinical severity, PCR testing, and spiral chest CT scanning. All surgical procedures were performed in accordance with the ethical principles outlined in the World Medical Association Declaration of Helsinki. The protocol was approved by the Ethical Committee of the Siberian State Medical University, and personal written consent for the upcoming intervention and potential concomitant risks including restenosis, recurrent laryngeal nerve plasty, tracheostomy, voice/swallowing dysfunction, etc. was obtained from each patient.

According to the rating scale for the degree of pulmonary damage, CT-3 was observed in two patients (18.2%) and CT-4 in seven cases (63.6%). In two cases, COVID-19 pneumonia with CT-2 lung tissue damage was accompanied by the initially established acute cerebrovascular accident of the ischemic type and epilepsy with a refractory status of tonic-clonic seizures.

Surgical procedures included circumferential tracheal resections in three patients and circumferential tracheal with partial cricoid resection in one patient. The length of excised trachea ranged from 20 mm to 35 mm (median 25 mm), and the number of rings resected ranged from two to eight (median five rings). Subhyoid laryngeal release was performed in one case.

An anesthesiologist, pulmonologist, and otolaryngologist who have completed a specialized training course in tracheal surgery were among the surgical team treating for CTS. The pivotal approach in CTS diagnosis was spiral computed tomography (CT) with multiplanar image reconstruction. In addition, a routine fibrobronchoscopy examination was conducted to evaluate vocal cords, glottis, cricoid and tracheal constituents. The length of the pathological area was measured, and its proximity to the vocal cords and the severity of stenosis were assessed using the Meyer-Cotton grading scale.

The operative approach was through a cervical collar incision in nine patients, whereas a cervical incision with sternal split was in one case and endoscopic in another. Dilation of CTS was performed in one case using rigid bronchoscopy in a patient who received a tracheal stent.

### 2.1 Tracheal resection

To reconstruct the tracheal and laryngotracheal lesion, we followed the surgical guidelines reported by Stoelben et al. [22] and Grillo [23]. Briefly, a patient is positioned supine on an operating table with a spine pillow superior to the mid border of the scapula, and the neck hyperextended. After the patient is anesthetized via an endotracheal tube inserted above the stenosis, the anterior surface of the trachea is exposed by the collar incision. In the case of inflammatory stenosis, dissection is mainly performed on the anterior surface of the trachea with particular emphasis on the lateral area(s) to exclude lesions to the vascular supply and the recurrent laryngeal nerves lying in the tracheoesophageal groove. Upward mobilization of the distal tracheal segment is achieved through blunt dissection of the anterior surface of the cervicomediastinal trachea.

In all cases except for the endoscopic procedure, the orotracheal tube was placed to allow a small-caliber tube to pass below the vocal folds. After excision, the distal trachea was intubated to allow anastomosis of the posterior suture line and removed with the passage of the orotracheal tube while performing the anterior suture line. If needed, the orotracheal tube was also used for bronchoscopy during transillumination, facilitating the demarcation of the superior and inferior margins of the cicatricial stenosis by atraumatic ligatures using interrupted sutures of 3-0 polyglactin (Vicryl, Ethicon, Inc., Somerville, NJ). Then, the trachea was divided below the stenotic area; the ventilation was performed with a cross-field endotracheal tube placed in the distal tracheal tract. The anesthetist withdrew the original orotracheal tube, and the end was tied up with sutures, so it could be drawn back before the anastomosis was completed.

**Fig. 1:**
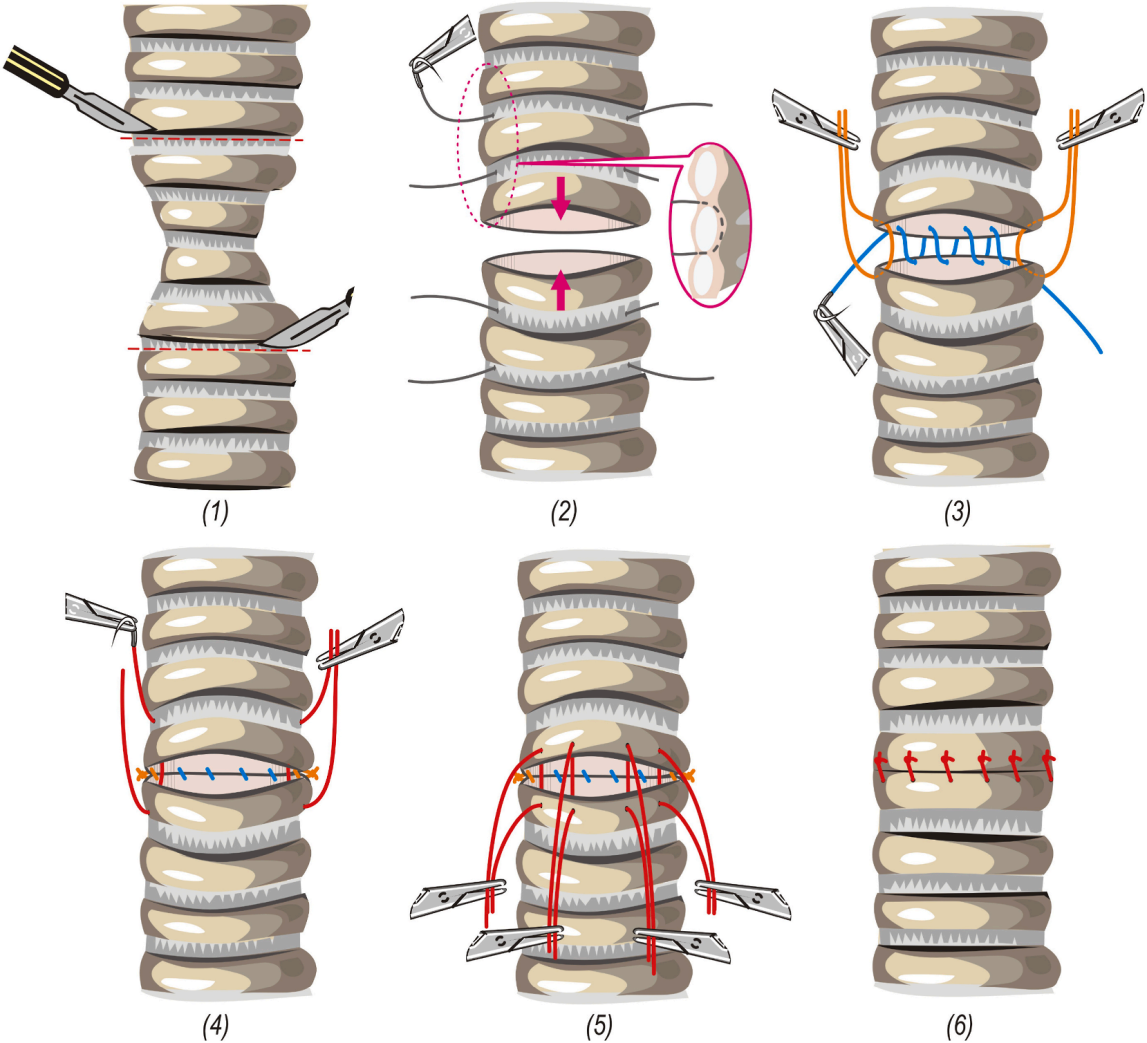
Stages of tracheal end-to-end anastomosis formation: (*1*) CTR of the stenosed tracheal area; (2) placing midlateral traction sutures (2-0 Vicryl, Ethicon); (3) placing the continuous 3-0 PDS suture; (*4*) the tightened continuous suture with placing lateral adaptive sutures; (*5*) placing interrupted sutures of 3-0 polyglactin (Vicryl, Ethicon); (*6*) view of the created anastomosis.

First, a continuous absorbable adaptive 3-0 PDS suture was posteriorly placed on the membranous part of the trachea without reducing or tightening the proximal and distal tracheal edges (Fig. 1). After applying the adaptive suture to the posterior wall of the anastomosis, separate sutures were laterally placed on the cartilaginous part of the trachea adjoining to the membranous part. Then, using the traction sutures (2-0 Vicryl, Ethicon), an assistant surgeon tightened and apposed the proximal and distal tracheal edges. The surgeon pulled on that continuous 3-0 PDS suture, thereby adapting the posterior wall of the anastomosis. Due to the behavior intrinsic to the PDS suture (high sliding ability, negligible capillarity), it is straightforward to reposition the tracheal edges, avoiding the rupture of the tracheal wall. After the edges were confirmed to match by visual control of the surgical wound, individual Vicryl sutures were laterally tied. Free PDS suture end was alternately bounded to these Vicryl sutures at the level of the lateral wall of the anastomosis. Separate interrupted sutures of 3-0 polyglactin (Vicryl, Ethicon) were placed at the lateral and anterior walls of the anastomosis.

During the anastomotic phase, ventilation was ensured from the cross-field tube. After the resection of the stenotic tract, the primary anastomosis was performed with a continuous suture of 3-0 polyglactin (Vicryl, Ethicon) for the membranous pars and with 8–10 interrupted sutures of 3-0 polyglactin (Vicryl, Ethicon) for the cartilaginous part. The orotracheal tube was then placed through the anastomosis, and all the knots of the sutures were tied outside.

### 2.2 Tracheal resection and dissection of tracheoesophageal fistula

Considering CTS combined with tracheoesophageal fistula (TEF), we planned circumferential tracheal resection (CTR) with the subsequent formation of the end-to-end tracheal anastomosis described above with sutured esophageal defect. Transtracheal access should be provided in this case. Given the TEF localization between the cervical and thoracic part of the trachea, collar cervical access only is preferred. Transillumination was used to determine a stoma of TEF and CTS. The margins of the upcoming resection area were demarcated using tracking surgical suture threads. CTR with leaving a fragment of the membranous wall of the TEF orifice was performed. Thus, a transtracheal route to the TEF area was provided with good visualization of the TEF orifice to be sutured, treating the patient with combined pathology in one procedure.

The trachea's cranial and caudal parts were very carefully and minimally dissected from the esophagus. The fistula was then sutured with a double-row continuous blanket atraumatic suture of 3-0 polyglactin (Vicryl, Ethicon) using a nasogastric tube (18 Fr). The first suture involved the membranous part of the trachea, whereas the second one used the esophageal muscularis mucosae, with the first suture dipped therein. The area of the sutured esophagus was rotated to seclude the tracheal and esophageal sutures. The integrity of the airway was then restored by applying end-to-end tracheal anastomosis, as depicted in Fig. 1.

After tracheal resection, the surgical area was drained in all cases using a 16 Fr tube through a separate incision in the left supraclavicular area. From the second day post-op, enteral nutrition was administered through a nasogastric tube (18 Fr) by drip infusion of a nutritional mixture.

### 2.3 Laryngotracheal reconstruction

Staged reconstructive plastic surgery consists of dissecting the stenosed laryngotracheal section of the scar tissues, obstructing the passage of air, excised from the lumen, followed by a reliable tracheostomy or tracheolaryngostomy technique. The airway lumen is subsequently formed within a few months of using a T-shaped silicone stent protector.

An X-shaped skin incision was made, and the skin flaps were mobilized. The anterior surface of the cervical trachea was isolated, focusing on the cricoid cartilage and the first tracheal semicircle. In functioning tracheostomy, the first surgical step was a vertical skin incision with excision of the tracheostomy canal and granulation tissue removal.

Then, craniocaudal incisions of the trachea were made to the level of intact cartilaginous semirings. In the case of circular obstruction, additional incisions in the tracheal wall in zones corresponding to the 4 and 8 o'clock positions are the safest ones. At this surgical phase, mechanical ventilation was carried out under visual control using an orotracheal tube passed through the glottis. Once the obstructed tracheal section has been dissected, making the respiratory tract free from the scarring tissues, the second surgical step was the formation of the stoma. The latter was performed by suturing the skin to the tracheal walls using interrupted sutures of 3-0 polyglactin (Vicryl, Ethicon), involving an encircling stitch on the wall of the trachea first. It was subsequently fixed to the skin. The tracheal mucous coat was precisely overlapped with the skin. The T-shaped stent was then inserted into the lumen of the respiratory tract.

Standard methods of biomedical statistics were used for statistical analysis of the obtained data using the software package StatSoft Statistica, ver. 10.0. In statistical analysis of the material, descriptive data are presented as mean (± standard deviation), median, and percentiles.

## 3 Results

Eleven working-age (34–68 y.o.) individuals were treated for cicatricial tracheal stenosis (CTS or PILS) after COVID-19 associated pneumonia and invasive mechanical ventilation (Table 1). Before tracheal intubation, all patients received respiratory support through noninvasive ventilation in a prone position, and this stage was stopped after changing ventilatory support for the invasive option. Post-tracheostomy stenosis was diagnosed in ten (90.9%) patients, whereas post-intubation stenosis − was in one (9.1%) case. Endotracheal intubation combined with a tracheostomy was performed in ten cases, whereas solely endotracheal intubation was in one case. Before surgical tracheostomy, all patients were intubated by an endotracheal tube, size 8, 8.5, or 9. Early open tracheostomy was performed in ten individuals who required prolonged tracheal intubation.

**Table 1:** Preoperative patient characteristics.

*No.*	Sex/age	Site of stenosis	CCES, mm	*Grade of stenosis	Malacia	TEF	Open tracheostomy	IMV, days	Comorbidities	Smoking	BMI	ASA
1	F/56	Multisegmental LTS	45	IV	Yes	–	Yes	28	Hypertension, Obesity	–	33.5	2
2	M/58	Cervical TS	25	II	–	–	Yes	24	Neurological deficit	–	26.4	3
3	F/50	Cervical TS	35	II	Yes	–	Yes	20	Gluteal abscess, DM, Neurological deficit, Hypertension, Obesity	–	37.2	4
4	F/51	Cervical TS	30	III	–	–	Yes	24	DM, Hypertension	–	22.2	2
5	F/34	Cervical TS	15	I	–	Yes	Yes	18	Epilepsy, Neurological deficit	–	22	2
6	F/43	Mediastinal TS	25	III	–	–	Yes	5	Hypertension	–	22.3	2
7	F/60	Cervical TS	40	III	Yes	–	Yes	130	Atrial fibrillation, DM, Neurological deficit, Hypertension, Obesity, Bedsores, Unilateral DP	–	41.5	4
8	M/61	Mediastinal TS	55	III	Yes	–	–	24	COPD, DM, IHD, Hypertension, Obesity	☑	43.5	4
9	M/55	Cervical TS	25	II	–	–	Yes	27	COPD Empyema of the pleura	☑	23.5	3
10	F/43	Cervical TS	40	III	Yes	–	Yes	9	Epilepsy, Hypertension, Obesity	–	31.5	3
11	F/68	Cervical- мediastinal TS	40	III	Yes	–	Yes	15	Atrial fibrillation, DM, Hypertension, Obesity, IHD, Bedsores	–	36.5	4
*Mean* (min-max)	52.64 ± 9.74 (34–68)	–	34.09 ± 11.36 (15–55)	–	–	–	–	29.45 ± 34.14 (5–130)	CCI mean = 5.18 ± 2.82 (2–10)	–	30.92 ± 8.08 (22–43.5)	3 ± 0.89 (2–4)

*Note*. LTS – laryngotracheal stenosis; TS - tracheal stenosis; CCES – the cranio-caudal extent of stenosis; TEF - tracheoesophageal fistula; DM - diabetes mellitus; COPD - chronic obstructive pulmonary disease; IHD – Ischemic heart disease; DP – Diaphragmatic paralysis; IMV - invasive mechanical ventilation; BMI - body mass index; CCI - Charlson comorbidity index; ASA – American Society of Anesthesiologists physical status classification system; * Myer-Cotton classification.

Retracheostomy at various terms after decannulation was performed in two patients. The duration of mechanical ventilation was from 5 to 130 days. The etiological factor of CTS determined its localization; therefore, in 82% of cases, it was located in the larynx and cervical trachea. Three patients were hospitalized with a functioning tracheostomy. In one case, extended CTS of the cervical trachea was combined with atresia of the laryngeal larynx and a Grade IV lesion of the left vocal fold. In one patient, post-tracheostomy CTS was combined with TEF. The length of the tracheal cicatricial changes ranged from 15 mm to 55 mm. One patient was admitted with stenosis Grade I and TEF, whereas Grade II and III were diagnosed in three and six individuals, respectively. Along with cicatricial changes in the tracheal wall, tracheomalacia was observed in 6 (54.5%) patients.

However, in our cohort, CTS detection in two (18.2%) patients had certain difficulties, and the correct diagnosis was constrained. First, some patients had a serious condition with severe polyneuropathy; they were in the intensive care unit with constant oxygen insufflation and were graded as ASA-IV at the time of CTS diagnosis. CTS in these individuals was accompanied by tracheomalacia; after decannulation, they needed a retracheostomy. Second, extensive lung tissue damage and developed complications induced poor post-COVID recovery dynamics, persistent dyspnea, and respiratory failure. Besides, one patient was also diagnosed with total relaxation of the right-sided diaphragmatic dome, which was not evidenced before infection with the SARS-CoV-2 virus.

In our cohort, for the etiotropic treatment of COVID-19, all patients received antiviral Remdesivir, strictly adhering to the recommended regimen. Given a severe course of COVID-19 infection (pneumonia with respiratory failure, acute respiratory distress syndrome), for the pathogenetic treatment, all patients received parenterally low molecular weight heparin (Nadroparin calcium) in a therapeutic dose and glucocorticosteroids (Dexamethasone) in the dose of 16–24 mg/day intravenously. It was the maximum dose prescribed for 3–4 days. Afterward, the DEX dose was reduced by 20–25% per injection every 1–2 days and then by 50% every 1–2 days until complete withdrawal. Since the patients were administered glucocorticosteroids, a treatment plan also included an interleukin (IL)-6 receptor blocker (Artlegia, R-Pharm), adhering to the prescribed regimen.

Antibacterial therapy was administered if obvious signs of secondary bacterial infection were noticed. The primary choice of antibacterial drug stemmed from the pneumonia severity, followed by correction after microbiome tests of expectorated sputum from the tracheobronchial tree. Once signs of purulent complications (gluteal abscess, empyema of the pleura, bedsores, sepsis, etc.) were noted, we withdrew glucocorticosteroids, selecting antibiotics according to bacteriological cultures from the purulent focus, coordinating our curative tactics with a clinical pharmacologist.

**Fig. 2:**
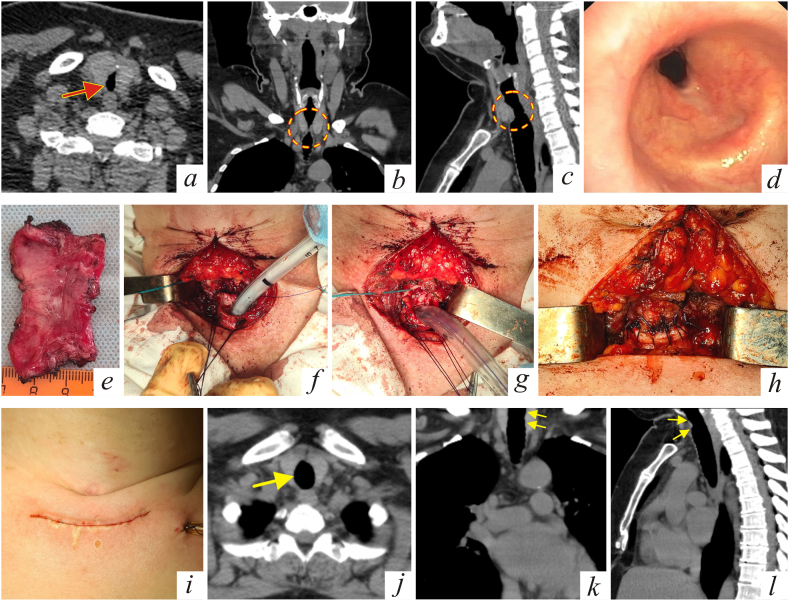
Typical patient (female, 50 y.o.) from our cohort who developed post-resuscitation CTS after COVID-19 associated pneumonia: pre-op (*a*) axial, (*b*) coronal, and (*c*) sagittal computed tomography (CT) scans and (*d*) bronchoscopic image showing tracheal lesion; (e*-i*) intra-op images exemplifying the excised tracheal fragment and surgical stages of end-to-end anastomosis formation according to Fig. 1; (*j-l*) post-op CT scans in six months, where yellow arrows indicate the consistent anastomotic area. (For interpretation of the references to colour in this figure legend, the reader is referred to the Web version of this article.)

At the preoperative stage, all patients were diagnosed with one or more severe concomitant diseases, and two individuals among them were smokers. The average value of the preoperative status according to the Charlson comorbidity index was 5.18 points, and the average body mass index in the cohort was 30.92. The physical status, according to the classification of the American Society of Anesthesiologists, was assessed in 4 (36.4%) cases as ASA-II, in 3 (27.2%) cases as ASA-III, and in 4 (36.4%) cases as ASA-IV. To restore airway patency, the CTR procedure was performed with anastomosis from the cervical and cervico-mediastinal access (Fig. 2), laryngotracheoplasty as the first phases of staged reconstructive plastic surgery (SRPS), along with endoscopic procedures (Table 2, Table 3).

**Table 2:** Features of the surgical treatment.

*No.*	Approach	LES, mm	Ext.	T-tube, month	Stent, month	Operative time, min	Noted complication	PLoS, days	Outcome
1	Cervical	0	OR	8	–	50	–	15	Good
2	Cervical	25	IPO	–	–	110	–	9	Excellent
3	Cervical	0	24 h	12	–	50	–	26	Excellent
4	Cervical	30	2 h	–	–	120	–	10	Excellent
5	Cervical	35	2 h	–	–	130	–	13	Excellent
6	Cervical/PS	20	3 h	–	–	205	–	7	Excellent
7	Cervical	0	72 h	3	–	45	PFSTS	23	Satisfactory
8	Endoscopic	0	OR	–	6	40	Stent migration, restenosis, tracheobronchitis	12	Not satisfactory
	Cervical	0	1 h	3		75	–	24	Good
9	Cervical	0	IPO	3	–	45	–	18	Excellent
10	Cervical	0	IPO	6	–	50	Vocal fold synechia	18	Good
11	Cervical	0	IPO	3	–	60	–	11	Excellent
*Mean* (min-max)	–	27.5 (20–35)	–	–	–	82.3 ± 52.7 (40–205)	–	14.7 ± 6 (7–26)	–

*Note*. PS – partial sternotomy; LES – length of the excised segment; Ext. – extubation; OR – in the operating room; IPO – immediate postoperative period; PLoS – postoperative length of stay; PFSTS – partial failure of the skin-tracheal suture.

**Table 3:** Options for surgical interventions for CTS.

Option	*No.*
CTR with anastomosis	3
CTR with anastomosis and separation of the tracheoesophageal fistula	1
Laryngotracheoplasty with T-stent endografting	7
Endoscopic stenting using a Dumont-type stent*	1
*Total*	12

*Note.* * - one patient required a re-stenting procedure due to stent migration, and granulation tissue with signs of purulent tracheobronchitis was observed in the trachea. We successfully performed laryngotracheoplasty with a T-tube stent.

The outcome was classified as excellent, good, satisfactory, or not satisfactory and was analyzed at the time of hospital discharge and after three and six months, respectively. The results were classified as excellent if the voice and respiration were completely routine and radiological examination, bronchoscopy, or both demonstrated an airway that was essentially normal in diameter. Good results were judged in the presence of a slight lessening of the maximum vocal volume, slight hoarseness, slight weakness of voice after prolonged use, and adequate breathing for everyday activities. Results were classified as satisfactory in the case of a hoarse voice with slight wheezing or shortness of breath on exercise, not sufficient to impair normal activities. Results were unsatisfactory, at the hospital discharge, in case of major complications [3]. We had no intraoperative mortality.

One patient with СTS underwent circumferential resection of the cervical trachea with excision of the arch of cricoid cartilage with tracheoglottic anastomosis. In the other two cases of circumferential resection of the cervical and thoracic trachea, the integrity of the airways was restored by an intertracheal anastomosis. In the case of CTS in combination with TEF, CTR was performed with the restoration of the respiratory lumen by applying intertracheal anastomosis and uncoupling the pathological anastomosis.

Histopathological examination of the resected airway demonstrated typical findings of post-intubation tracheal injury, including mucosal ulceration, squamous metaplasia, stromal fibrosis, and mixed acute and chronic inflammatory infiltrates.

Primary laryngotracheoplasty using a T-shaped silicone stent was performed in six patients. In these cases, the lesion level was limited to the laryngeal lining and cervical trachea, whereas that belonged to the cervicothoracic area in one case. Therefore, the stoma was applied in the area of stenosis. It maintains the consistency of the intact trachea, allowing local treatment and removal of granulations. The length of the procedure is around 50 min, and its invasiveness is not significantly different from that of a routine tracheostomy, which does not worsen the post-operation general condition of the patient. The post-operation period in one patient from the cohort was complicated by the partial inconsistency of the skin-tracheal suture. Comprehensive care, including antibiotic therapy and daily dressings with stent patency control, allowed this complication to be conservatively managed. Notably, according to this patient's medical file, the tracheobronchial tree's obstruction resulted not only from CTS but also from an abundant volume of viscous sputum.

One patient with extensive lesions of the thoracic trachea with tracheomalacia, having no functioning tracheostomy, underwent tracheal bougienage and received a silicone Dumont-type stent. However, on the fourth day after stenting, caudal displacement of the stent was noted, which required revision and re-stenting with a favorable outcome. However, 11 months later, we emphasized the development of granulation restenosis along the stent cranial margins to the extent of the subclavian part of the larynx, accompanied by purulent tracheobronchitis, respiratory failure, decreased saturation, and stridor at minimal physical load. Endobronchial granulations could not be removed, and we decided to extract the stent and successfully performed the laryngotracheoplastic procedure using a T-shaped stent.

Considering this case and the early tactics, it can be argued that endoscopic stenting was due to several reasons, including a rational concern for performing the CTR procedure and high risks of tracheal suture inconsistency. We took into account the length of the tracheal lesion and anatomical features - short and almost immobilized neck, and the presence of multimorbidity. The difficulty in performing laryngotracheoplasty and subsequent medical care of the T-stent could not be ignored either. Besides, the patient categorically refused the laryngotracheoplastic procedure, preferring endoscopic tracheal stenting using a Dumont self-fixing stent. Of course, we had doubts concerning the success of tracheal patency restoration in this particular case, which can be explained by little clinical experience in treating PITS after COVID-19 associated pneumonia.

Analysis of patients' outcomes showed that antivirals, high doses of glucocorticosteroids, and direct therapeutic doses of anticoagulants predominated in the treatment of COVID-19 associated pneumonia. The curative algorithm for CTS after airway patency restoration surgery included a session of hyperbaric oxygenation compulsorily to assist with anastomotic healing based on our early experience. For example, even in the small series, there may be some advantages to the availability of hyperbaric oxygen treatment.

All patients tolerated the suggested surgical treatment well and managed to restore adequate breathing through the natural airways with preserved vocal function. There were no lethal outcomes in the post-op period. Clinical outcomes in four patients with CTS after CTR are assessed as excellent, as all these individuals fully recovered and returned to work and normal life. In three patients after laryngotracheal reconstruction who completed full treatment, the results of treatment were also excellent, and in three patients, the results were good. One patient is still in the final phase of recovery after laryngotracheoplasty.

## 4 Discussion

As we study the risk factors and consequences of SARS-CoV-2 infections, the emergence of a new category of patients seems to be obvious. This cohort has distinctive features in diagnosis and treatment tactics, including post-intubation tracheal after-effects and complications. Currently, tracheomalacia, CTS, and TEF or their combinations are considered to be among frightful and challenging sequelae [5,7,[24], [25], [26]].

Overpressure due to an overinflated cuffed endotracheal or tracheostomy tube within the context of microcirculation disorders leads to local ischemia in the tracheal wall, mucosal edema/hemorrhages, chondroperichondritis, necrotic and sequestrated tracheal cartilage with subsequent substitution with granulation and coarse connective tissue. These pathological processes are often the cause of CTS. In some cases, the morbid reaction in the trachea occurs, resulting in degeneration of the bronchial epithelium, evidenced in the form of erosions, ulcers, and tracheoesophageal fistulae stemming from transmural necrosis [23].

Clinical reports suggest that COVID-19 patients experience extended stays in the ICU with prolonged intubations, stretching from one to two weeks or longer [27]. In another published report [17], one patient required five days and the other 130 days of ventilation. Although laryngotracheal stenosis is associated with prolonged mechanical ventilation, we herein report cases representing both relatively short and prolonged intubations with a tracheostomy. The length of time to the onset of CTS varied on a case-by-case basis. Factors associated with CTS development include prolonged intubation, tracheal trauma during intubation, size and quality of the endotracheal tube, an overinflated tracheal tube cuff and its displacement after intubation. Li et al. previously reported that orotracheal intubation over ten days before tracheostomy was associated with increased rates of CTS [6]*.* Ershadi et al., in turn, reported a patient with confirmed COVID-19 but without a history of tracheal intubation diagnosed with distal tracheal stenosis [28].

To date, international guidelines for tracheostomy in COVID-19 patients and their subsequent management have not yet been accepted. Regardless of the fact that we used a standard cuff pressure and appropriate tube sizes, we experienced 11 cases of PILS in patients who recently underwent mechanical ventilation due to COVID-19 in a relatively short period.

There was no evidence to suggest that the observed damage to the larynx and trachea was directly caused by COVID-19. However, in COVID-19 patients, long-term intubation and a persistent cough may cause laryngotracheal mucosal damage and inflammation, leading to tracheal stenosis. Furthermore, obesity was associated with increased rates of developing post-intubation tracheal stenosis [29]. Co-morbidities also play a role – six patients had diabetes and a high body mass index (BMI), constituting risk factors for tracheal stenosis. Laryngopharyngeal reflux occurs in obese and intubated patients, and the resultant chronic inflammation is associated with subglottic stenosis. A high BMI will also compound airway problems associated with OSA and laryngo-tracheomalacia. In our cohort, we noted a high BMI in all six cases.

In general, the development of CTS is believed to be revealed by careful analysis of complaints from the patients, a history of invasive mechanical ventilation, and distinctive stridor breathing. A diagnostic approach has been developed to detect CTS, and the pivotal methods to check this pathology are CT scanning and fibrobronchoscopy examination, which seem to exclude diagnostic errors. The most common and frequently reported symptoms of COVID-19 are fever and cough. A solid and persistent cough, in addition to increased inspiratory effort, may pose an inherent risk of developing PILS after prolonged intubation. It should be noted that the clinical manifestations of PILS closely resemble those of severe COVID-19 in the recovery phase.

In this category of patients with minimal suspicion of CTS, it is necessary to obtain CT scans of the neck and chest organs with particular emphasis on the trachea, as depicted in Fig. 2. A radical surgical method that treats both CTS and TEF is circumferential resection of the cicatricial trachea, separation of tracheoesophageal fistula with the restoration of the consistent tracheal wall, and esophagus [26,30,31]. Tracheal resection is, however, difficult from a practical viewpoint and has strict indications; it can be related to life-threatening intra- and post-op complications. Technically, staged reconstructive plastic surgery is considered to be routine and easier to perform. Despite its simplicity, this procedure can also be characterized by major shortcomings in the medium and long terms [3,5,31].

Interventional bronchoscopy role, such as mechanical dilatation, laser ablation, and stenting, is limited since the recurrences are frequent and usually reserved for palliative endoscopic laser treatment. Endoscopic treatment of complex stenosis extended over 1 cm and with tracheal wall involvement is contraindicated, and when feasible, surgery should remain the treatment of choice. In fact, mechanical dilation for complex stenoses leads to a recurrence rate of >90% [32].

Our case series of treated CTS after COVID-19 associated pneumonia demonstrates very low effectiveness of endoscopic bougienage and stenting of the obstructed trachea. Trachea lesion is assumed to be due to extended tracheomalacia and the characteristic features of the tracheal wall. Although there are reports with optimistic outcomes, e.g., Gervasio et al. [16] reported managing cases of tracheal stenosis following COVID-19 with intravenous corticosteroids could have an appropriately salutary effect. However, in our observation, even though all patients received high doses of corticosteroids, they developed CTS. Post-tracheostomy stenosis deformity usually responds to bronchoscopic interventions, but this treatment does not appear to be as responsive in COVID-19 patients.

The optimal time and type of surgical approach for post-resuscitation CTS aggravated with TEF after COVID-19 associated pneumonia has not been suggested thus far. In the acute stage of coronavirus infection, CT scans indicate that the extent of lung damage correlates with the severity of the disease. In all patients, thoracic CT check before surgery revealed remaining signs of developed coronavirus pneumonia, evident as numerous areas of consolidated and compacted patterns of various shapes and lengths, similar to frosted glass texture. The permanent radiological patterns after suffering from coronavirus pneumonia are individual and do not have a clear resolution period. It has been shown that after a severe course of the disease, changes of varying severity can persist for up to 6 months [13].

Muscle weakness and fatigue, asthenic syndrome, and anxiety-depressive disorders of varying severity were observed in all patients and are indicative of post-covid syndrome. In such patients, polyneuropathy is inherent due to both covid infection and critical conditions. Relaxation of the diaphragm is assumed to be also a manifestation of post-covid polyneuropathy, which significantly aggravates the patient's condition due to respiratory failure. In addition, polyneuropathy as a complication of comorbid pathology in the form of type 2 diabetes mellitus in five (45.5%) patients also had an adverse effect.

Depending on the general condition, the extent of narrowing, and the length of CTS, the period from the diagnosis to the surgical procedure varied from one day to seven weeks. In cases where it was possible, surgical treatment was postponed due to the high risk of current complications, and a course of pulmonary rehabilitation was carried out with subsequent CT monitoring of the chest organs. Also, a multidisciplinary approach needs to include inputs from a thoracic surgeon, anesthesiologist-resuscitator, endoscopist, and pulmonologist. The team makes the panel decision on the time and type of surgery aimed at restoring the patency of the airways. The determining factor was the preoperative physical and functional status of the patient, which was matched with the surgical postulate in the treatment of CTS and TEF. In so doing, the most radical surgery with a high quality of life is CTR.

## 5 Conclusions

COVID-19 patients who underwent invasive mechanical ventilation, regardless of its duration, are at high risk of developing CTS and require follow-up. To date, there are no official clinical guidelines for the treatment of post-resuscitation CTS after COVID-19 associated pneumonia. Our experience shows that CTR, when strict indications are established, allows a patient to be rehabilitated in a short time with the best functional outcomes.

If CTR is contraindicated, staged reconstructive plastic surgeries are recommended, which allow, along with restoring the tracheal lumen, to make adequate daily debridement of the tracheobronchial tree. Besides, the closed upper knee of the T-shaped stent maintains respiratory support through invasive mechanical ventilation. Our case series shows that these patients can present with pathologies at multiple levels in the subglottis and trachea, requiring multiple interventions for recurrent stenosis.

Intraluminal endoscopic tracheal patency aids are an alternative to open surgery, especially for intrathoracic CTS and intractable tracheobronchitis. Further studies of this pathology and clarification of indications for specific types of surgical interventions to restore the airway lumen are required.

## Funding

This work was supported by the 10.13039/501100003443Ministry of Education and Science of the Russian Federation, project No. FSWM-2020-0022.

## Ethics approval

Approval from an institutional board review was received.

## Animal rights

This work does not contain any studies with animals performed by any of the authors.

## Informed consent

Written informed consent was obtained from each patient before surgical intervention.

## Availability of data and material

All data are available from authors upon reasonable request.

## Code availability

N/A.

## Consent for publication

N/A.

## Authors’ contributions

Conceptualization, methodology, investigation, E.T.; validation, formal analysis, visualization, T.C. and E.M.; writing - original draft preparation, T.C.; writing - review and editing, A.V.; project administration, E.M.; funding acquisition, E.M. and A.V. All authors made substantive edits for critical content and approved the final manuscript.

## Declaration of competing interest

The authors declare that they have no known competing financial interests or personal relationships that could have appeared to influence the work reported in this paper.
